# The acute medical unit model: A characterisation based upon the National Health Service in Scotland

**DOI:** 10.1371/journal.pone.0204010

**Published:** 2018-10-03

**Authors:** Lindsay E. M. Reid, Ursula Pretsch, Michael C. Jones, Nazir I. Lone, Christopher J. Weir, Zoe Morrison

**Affiliations:** 1 Development and Delivery Department, Ko Awatea Health Systems Innovation and Improvement, Auckland, New Zealand; 2 Quality, Research and Standards, Royal College of Physicians of Edinburgh, Edinburgh, United Kingdom; 3 Usher Institute of Population Health Sciences and Informatics, University of Edinburgh, Edinburgh, United Kingdom; 4 Edinburgh Clinical Trials Unit; University of Edinburgh, Edinburgh, United Kingdom; 5 Business School, University of Aberdeen, Aberdeen, United Kingdom; University of South Australia, AUSTRALIA

## Abstract

**Background:**

Acute medical units (AMUs) receive the majority of acute medical patients presenting to hospital as an emergency in the United Kingdom (UK) and in other international settings. They have emerged as a result of local service innovation in the context of a limited evidence base. As such, the AMU model is not well characterised in terms of its boundaries, patient populations and components of care. This makes service optimisation and development through strategic resource planning, quality improvement and research challenging.

**Aim:**

This study aims to evaluate a national set of AMUs with the intent of characterising the AMU model.

**Methods:**

Twenty-nine AMUs in Scotland were identified. Data were collected by semi-structured interviews with multidisciplinary healthcare professionals working in each AMU. A draft report was produced for each unit and verified by a unit representative. The unit reports were then analysed to develop a conceptual framework of key components of AMUs and a service definition of the boundaries of acute medical care.

**Results:**

Acute medical care in Scotland can be described as being delivered in “acute medical services” rather than geographically distinct AMUs. Twelve key components of AMU care were identified: care areas, functions, populations, patient flow, support services, communication, nurse care, allied healthcare professional care, non-consultant medical care, consultant care, patient assessment and specialty care.

**Discussion:**

This empirically derived characterisation of the AMU model is likely to be of utility to practitioners, managers, policy makers and researchers: it is relevant on an operational level, will aid quality improvement and is a foundation to needed further research into how best to deliver care in AMUs. This is important given the central role AMUs play in the journey of the majority of patients presenting to hospital acutely in Scotland, the UK and internationally.

## Introduction

Acute medical units (AMUs) have been defined by the Royal College of Physicians in London (RCP) as “a dedicated facility within a hospital that acts as the focus for acute medical care for patients who have presented as medical emergencies to hospital”[1]. “The acute medical care delivered in AMUs encompasses that provided by a physician to patients with medical complaints (such as heart attacks, strokes, and pneumonia). It does not include care provided to paediatric, psychiatric, surgical or obstetric/gynaecology patients. Patients enter AMUs by referral from either the Emergency Department (ED) or a General Practitioner (GP) in the community. As such, AMUs are located at the interface between ED/ GP and formal hospital based care that is usually delivered in inpatient medical specialty wards (such as a respiratory ward). AMUs differ from EDs in that they only deliver care to patients with acute medical presentations, whereas EDs provide emergency care to patients with all types of presentation (such as those listed above). AMUs provide care to patients for a variable period of time following presentation to hospital, usually up to 72 hours.

AMUs emerged as a result of local service innovations in the United Kingdom (UK) in the 1990s. Prior to this, the traditional model for unscheduled medical care involved the admission of patients to multiple medical wards. This model was challenged as unfit for purpose by a number of professional bodies, including the Royal College of Physicians of Edinburgh (RCPE) and the Royal College of Physicians and Surgeons of Glasgow (RCPSG). They produced a joint report in 1998 advocating designated geographical areas within hospitals to receive medical patients from the ED and/or directly from the community. A number of hospitals developed such systems independently during this time, and the movement was formally recognised by the formation of the Society for Acute Medicine in 2000[2]. In 2003 Acute Internal Medicine was recognised as a sub-specialty in the UK and latterly as a specialty in its own right in 2009(2). A seminal report detailing recommendations of how to deliver care in AMUs was published by RCP in 2007[1].

During this time, the AMU model of care was progressively adopted throughout the UK. In Scotland, AMUs had been instituted in the majority of hospitals receiving acute medical patients by 2003[3]. In 2009 it was reported that 75% of patients were admitted directly to AMUs in the UK[4]. The AMU model has also been established outside of the UK: a report published in 2004 in Ireland advocated the AMU as the recommended model of care and led to its adoption by a number of Irish hospitals[5]; recent surveys have shown that the AMU model serves 42% of the New Zealand population[6]; 70% of hospitals responding in an Australian survey had established AMUs[7]; and there has been adoption of the AMU model in a number of European countries, including the Netherlands, Italy and in Scandinavia[8–10].

Despite this widespread adoption of the AMU model, the evidence base relating to the AMU is limited: our systematic review evaluating the effectiveness of the AMU model in comparison to alternative models of care identified just 17 studies of 12 AMUs[11]. This review found limited, observational and possibly confounded evidence that the AMU model was associated with reductions in hospital length of stay and mortality compared to other models of care in European and Australasian settings. This systematic review also found that there was variation in care delivery across AMUs in different setting, which is in keeping with other survey evidence [12–14]. This prompted a further systematic review of evidence for how best to deliver care within AMUs. Ten studies of nine interventions were identified and the review concluded that there was little discernible evidence to direct care delivery to optimise outcomes[15].

Because of the organic development of AMUs in the context of this limited evidence base, the AMU model is not currently well characterised. This has implications for optimising and developing the delivery of care in AMUs through resource planning, quality improvement including benchmarking and empirical research to inform how best to deliver care in AMUs. Such endeavours require a definition of the boundaries, patient populations and components of care in AMUs. Therefore, this study aims to evaluate a national set of AMUs to provide an evidence-based characterisation of the AMU model.

## Materials and methods

### Study design

**Fig 1:**
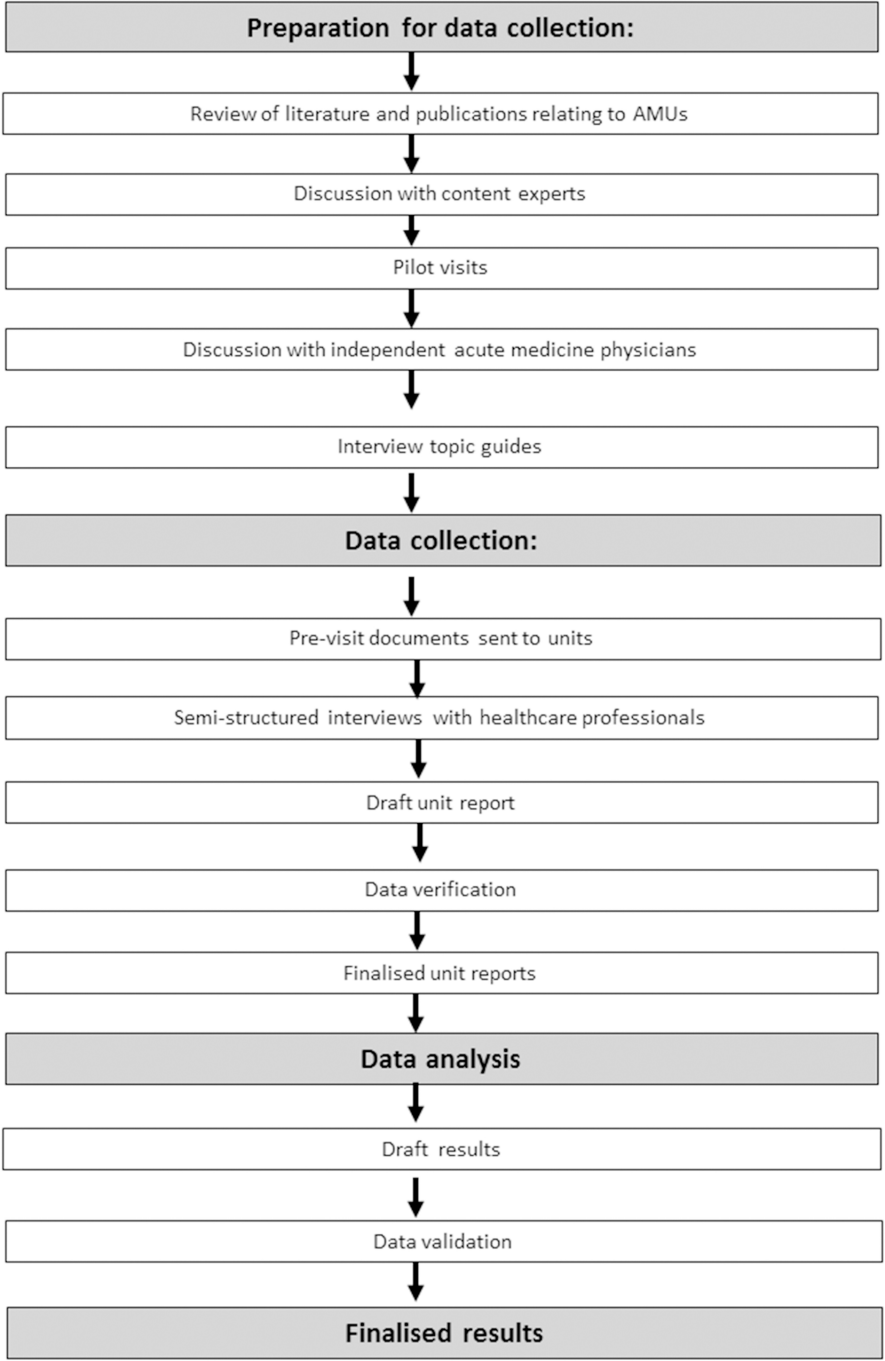
Summary of study design. AMU–acute medical unit.

A cross-sectional survey was conducted through a visit to each AMU in Scotland to undertake semi-structured interviews with a range of healthcare professionals. A flow chart depicting the study design is provided in Fig 1. The methodological orientation of this study is content analysis[16].

### Ethical considerations

The Ethical Committee in Centre of Population Health Sciences (CPHS) at the University of Edinburgh granted ethical approval for this work (date 17/03/2014). The need for formal ethical approval was waived by the Scientific Officer in the National Health Service (NHS) South East Scotland Research Ethics Service secondary to this work being classed as service evaluation (Reference: NR/1310AB25). All data were managed in accordance with the Data Protection Act 1998[17].

### Study setting and sample

Sampling was purposive and the study population comprised all AMUs in Scotland (*n* = 29). AMUs were identified through a review of hospitals that had facilities congruent with the RCP definition of an AMU[1]. This review was undertaken through discussions with healthcare professionals working in acute hospitals across Scotland and was cross-checked with information held by Information Services Division (ISD) Scotland (a branch of National Services Scotland that provides data support to National Health Service Scotland).

Participants were identified in each AMU as healthcare professionals fulfilling one of the following roles: nursing staff, allied healthcare professional staff, non-consultant medical staff and consultant medical staff.

### Study period

The period of data collection was from 15^th^ January 2014 to 22^nd^ March 2015.

### Recruitment

One representative from each unit was identified from members of an acute medicine working group hosted at the RCPE. These representatives were approached by email to coordinate the visits and identify potential interview participants for voluntary participation in the data collection.

### Data collection

Data were collected by semi-structured interviews. Although we considered the use of more structured data collections methods, such as a written survey, we judged that such methods would not adequately answer the research question: given the lack of *a priori* knowledge, the variation in care delivery across AMUs and the complexity of the subject matter, semi-structured interviews were selected to allow for a sufficiently nuanced insight into service delivery at each site.

The participants were emailed a participant information sheet detailing the rationale and process of the interviews prior to the unit visit. This was relayed verbally at the start of each interview with opportunity for the participants to seek clarification/raise any queries. Written consent was then obtained from each interview participant.

All interviews were conducted by the same researcher (LR) and observed by a colleague (UP) in accordance with principles of Good Clinical Practice[18, 19]. This provided both clinical (LR) and managerial (UP) insights into the data generation. The interview topic guides (Table 1) were developed in three stages. The first stage involved a review of the literature[11] and the existing recommendations for AMU care[1, 20, 21]. This was further informed by the clinical experience of the research group and through discussion with content experts, lay representatives and government representatives. In the second stage, the preliminary interview topic guides were refined iteratively during three pilot site visits that were undertaken prior to the start of data collection. This involved feedback from the healthcare professionals participating in the interviews. The selection of units used as pilot sites was based upon where the research team had pre-existing contacts in addition to ensuring the units included different health boards and a mix of hospital size. The pilot units were re-visited during the formal data collection stage using the same approach as with the other units. In the third stage, feedback on the topic guides was requested from three independent acute medicine physicians who had no formal involvement in the project.

**Table 1:** Interview topic guide.

Theme	Topic	Subtopics	Interview^1^
Physical	Context	Board context. Hospital context: • Number of medical beds and receiving wards. • ED/Surgical/Medical/O&G/Paediatrics services. • Provision of tertiary medical services.	Nurse 1
	Physical areas	Constituent parts including bed numbers and functions. Other areas of note (ED, HDU, stroke unit, CCU, day medicine unit). Geographical layout.	Nurse 1
	Facilities	Level 0/1/2 care. Monitoring–bedside, central, telemetry. Cohorted patients. Provision for central lines/arterial lines. EWS used. Care of the deteriorating patient.	Nurse 1
Process	Patient entry sources	ED/GP/other. Alternative entry routes for medical patients.	Trainee 1
	Specific presentations	DVT, time critical presentations such as patients requiring emergency percutaneous coronary intervention or stroke thrombolysis.	Trainee 1
	Patient journey	Referral/arrival/diagnostics/assessment/disposal.	Trainee 1
	Return and planned care	-	Consultant 1
	Alternatives to admission	-	Consultant 1
	Clinics	Provision for acute medical clinics.	Consultant 1
Procedural	Capacity management	Management of patient flow. Procedures at times of excess demand.	Nurse 1
	Communication	Safety briefs/board rounds +/- the multidisciplinary team.	Nurse 2
	Interfaces	Primary care, ED, critical care.	Trainees 1
Personnel	Nurse staffing	Role of nurse in charge. Staffing numbers (weekday/weekend/overnight, registered and unregistered).	Nurse 2
	Multidisciplinary staffing	Hours of cover (week/weekend). Responsibilities within and outside the AMU. Role.	Pharmacy; therapy.
	Non-consultant staffing^2^	Source. Grades. Hours and responsibilities within and outside the AMU (week/weekday/overnight). Downstream medical ward cover (evenings/nights/weekend).	Trainees 2
	Consultant staffing	Number of consultants at the week/weekend. Hours of presence. Responsibilities within and outside the AMU. Patient contacts. Other commitments. Source. Continuity. Frequency. Downstream cover. Direct clinical care programmed activity sessions within the acute medicine service.	Consultant 2
	Patient review	Frequency of review (direct/indirect, week/weekend).	Consultant 2
	Specialty care	Input to AMU per medical specialty (Care of the elderly, respiratory, cardiology, gastroenterology, diabetes/endocrinology, renal, rheumatology, haematology, neurology, stroke, infectious diseases, dermatology, oncology, palliative care). Specialist nurse availability. Availability of mental health, surgery and critical care support.	Consultant 1

ACP–acute care physician; ED–emergency department, O&G–obstetrics and gynaecology; HDU–high dependency unit; CCU–coronary care unit; EWS–early warning system; DVT–deep vein thrombosis, MDT–multidisciplinary team.

^1^Refers to which of the six interviews these topics were covered in.

^2^Non-consultant staff were defined as practitioners providing a medical role who were not acting at the level of a consultant. A medical role was defined as activities that related to the diagnosis and treatment of illness that are traditionally undertaken by doctors and that are separate to the activities of patient care traditionally undertaken by nursing staff.

The topic guides comprised four main themes: physical, process, procedural and personnel. The ‘physical’ theme included the geography, constituent parts and facilities of the AMU; the ‘process’ theme included detail of how patients proceeded through their care journey; the ‘procedural’ theme included detail on the operating procedures of the AMU; and the ‘personnel’ theme included staffing in the AMU.

During the data collection, the topics covered in each interview were applied flexibly to allow for possible disruptions, facilitate triangulation and allow clarification as required. Detailed field notes were taken by the interviewer (LR) and observer (UP) and interviews with nursing and medical staff were audio recorded. The interviewer’s (LR) field notes were used to generate a report for each unit contemporaneously to the unit visit.

### Data verification

Draft unit reports were checked by the observer (UP) and any discrepancies were resolved by discussion, by listening to the interview audio-recordings, or seeking clarification from the unit. Following this, a draft report was returned to the unit representatives for participant checking prior to being finalised.

### Data analysis

Data analysis was undertaken by a single researcher (LR) with regular discussion with a second researcher (ZM). Provisional deductive themes were identified in advance from the topics in the interview guides. These were then developed by iterative analysis of the unit reports to generate a conceptual framework of the key components of AMUs that accommodated all data in mutually exclusive categories. A key component was defined as an organisational factor that was integral to the operation of AMUs irrespective of the setting.

The unit reports were further analysed to define the boundaries of acute medical care using Ritchie and Lewis’s framework method[22]. In this method data are systematically indexed and charted to construct a two-dimensional matrix of cases down the vertical aspect of the matrix and categories across the horizontal aspect. A two-dimensional matrix was created with the units listed vertically and relevant data regarding the boundaries of the acute medical services (for example services offered and constituent areas) were added from each unit report. The matrix was then analysed to build a definition of acute medical care in Scottish hospitals. This method was chosen as it facilitated the inclusion of all units included in the study population and the systematic analysis of the boundaries of acute care at each site.

### Data validation

External input was sought from lay representatives, government representatives and content experts through the conception, preparation, execution and analysis of the study.

During the analysis phase, a meeting was held to discuss preliminary findings with a group of acute medicine and specialty physicians independent of the study to develop the analysis, check its validity and increase its objectivity. Similarly, also during the analysis phase, preliminary findings were presented for discussion at a national general medicine conference.

## Results

A total of 275 participants were interviewed, comprising 58 nurses, 58 allied healthcare professionals (AHPs), 83 non-consultant medical staff and 76 consultants. No participants declined to participate. The interviews ranged from 37 to 86 minutes in duration. All 29 of the reports were verified by a healthcare professional working within the unit. In 27 units the report was reviewed by a medical consultant and in two units it was reviewed by a charge nurse. The reports were modified in accordance to the feedback from the representatives.

Although the sampling strategy was purposive to include a national set of AMUs, data saturation was reached during the data collection process.

### Characterising the acute medical unit model: A framework for the key components of care

**Fig 2:**
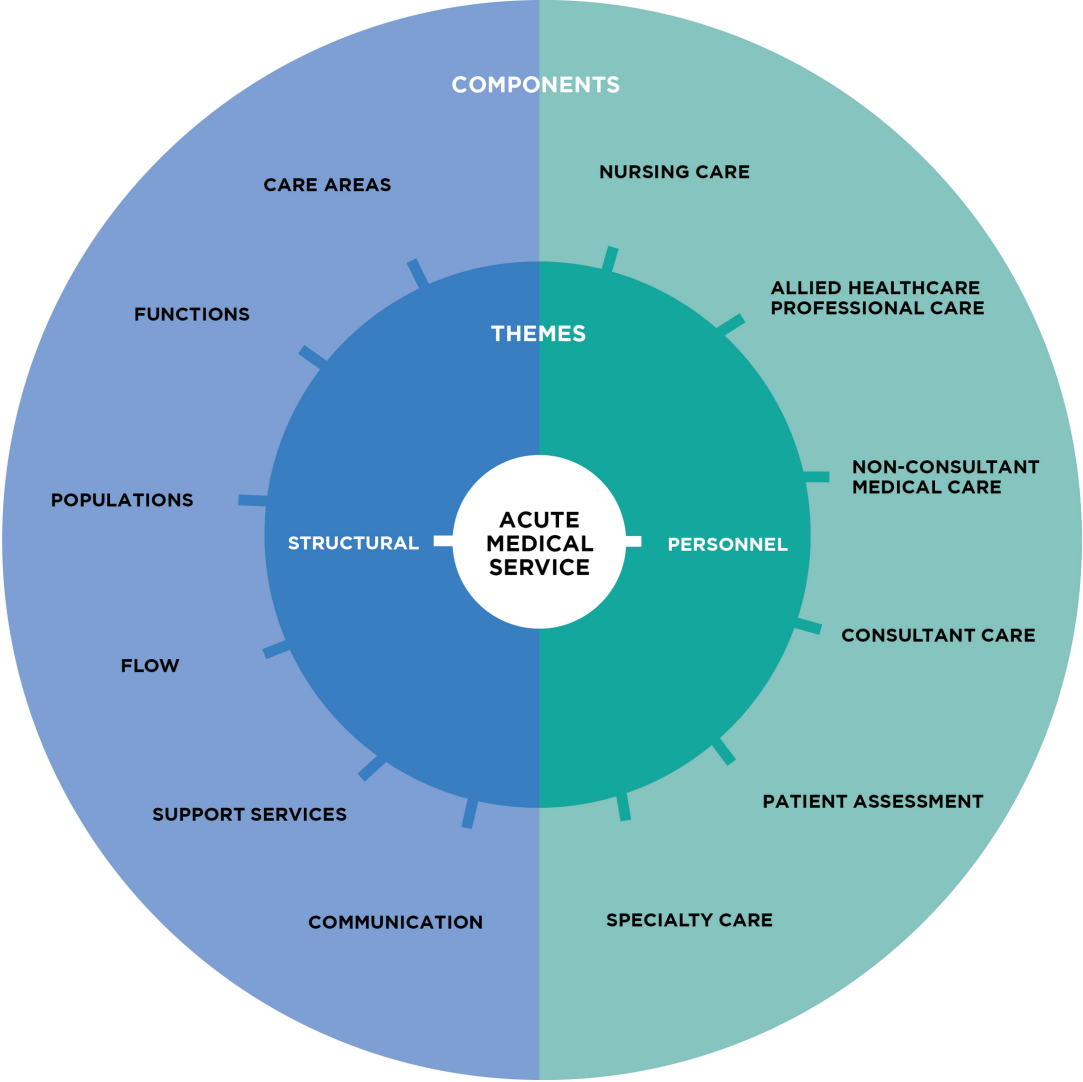
Key components of the acute medical service.

Twelve key components of AMUs were identified (Fig 2). These components were present in two main themes: i) structural and ii) personnel. The structural theme contained the following components: care areas, functions, populations, patient flow, support services, and communication. The personnel theme contained the following components: nurse care, AHP care, non-consultant medical care, consultant care, patient assessment and specialty care. The definition of each component is given in Table 2.

**Table 2:** Definitions of components.

Component	Definition
Care areas	Physically distinct areas of AMU.
Functions	The purpose of the activities undertaken as part of a patient’s care.
Populations	The patient types receiving care in the AMU.
Flow	“The movement of patients, information or equipment between departments, staff groups or organisations as part of the care pathway” [23].
Communication	Formal methods of communication between healthcare professionals groups in the AMU, including safety briefs and handovers.
Support services	The infrastructure of the ancillary services provided to patients in the AMU that are required for its operation. Includes services such as laboratories, radiology and information technology.
Nursing care	The provision of nurse staffing in the AMU. This excludes nurses undertaking a clinical role. Clinical role is defined as activities that relate to the diagnosis and treatment of illness that are traditionally undertaken by doctors and are separate to the activities of patient care traditionally undertaken by nursing staff.
AHP care	The provision for AHP input on the AMU.
Non-consultant medical care	The provision of staff providing a clinical role who are not acting at the level of a consultant. Medical role is defined as activities that relate to the diagnosis and treatment of illness that are traditionally undertaken by doctors and are separate to the activities of patient care traditionally undertaken by nursing staff.
Consultant care	The delivery of routine core AMU care by consultant staff.
Patient review	The routine contact of clinical staff with patients in the AMU.
Specialty care	The input specialty teams provide to specialty-specific patients on the AMU (for example, the input of the respiratory service to respiratory patients).

AMU–acute medical unit; AHP–allied healthcare professional.

### Characterising the boundaries of acute medical care in Scottish hospitals: An acute medical service

We found that in 19 of the 29 sites acute medical care was being delivered in one or more areas in addition to the main bedded AMU area. We also found that undifferentiated acute medical patients were being cared for by ED teams rather than the acute medical team in six sites. Furthermore, in 21% of sites acute medical patients whose presentation related to a specific specialty bypassed the AMU and were admitted directly to that specialty ward. In light of these findings, we propose that acute medical care in Scottish hospitals is delivered in “acute medical services” rather than geographically distinct AMUs. Acute medical services are defined as: the suite of dedicated facilities within hospitals that are under the responsibility of the acute medical team and provide acute medical care to undifferentiated patients who have presented to hospital as a medical emergency. Acute medical care is defined as that provided by a physician to patients with medical complaints (such as heart attacks, strokes and pneumonia). Undifferentiated refers to the acute medical population that has not been segregated into medical subspecialties (such as cardiology, respiratory etc.). The acute medical team is defined as members of the multidisciplinary team led by a physician whose predominant role is delivering care to patients who have presented as medical emergencies to hospital.

Our definition can be further clarified by means of an example. In one unit, the main AMU contained 40 beds. In addition to this there was a separate area located remotely from the main AMU in which an acute medicine physician conducted the assessment and treatment of ambulatory care patients. Using the definition generated from this study, the AMS within that hospital contains both the main AMU and the ambulatory care area. This site also contains a short stay facility that cares for some acute medical patients such as those with simple poisonings. This area is under the care of the ED and therefore is excluded from the AMS based on our definition.

## Discussion

Through this national evaluation of AMUs in Scotland, we present an evidence based characterisation of the AMU model. This is the first detailed examination of this topic, which is based upon a robust description of acute medical practice across and entire country. Our findings are an important resource for understanding the acute medical model of care which is an essential step in improving the quality of care delivered to acute medical patients.

Firstly, we provide a definition of the boundaries of the AMU model: acute medical care in Scotland can be described as being delivered in acute medical services rather than geographically distinct AMUs. This definition represents an update of the currently accepted RCP definition. Our definition describes a “suite of dedicated facilities” as opposed to the “dedicated facility (…) which acts as a focus of acute medical care” referred to in the existing definition. This updated definition has the advantage of capturing acute medical care activity in its totality and is a means of defining the patient population of interest.

This is relevant on an operational level given that an accurate definition of acute medical activity will allow more accurate resource allocation and governance, both locally and nationally. It is also necessary to facilitate quality improvement: individual AMUs wishing to use data to undertake a service review and improvement work require a means of delineating the boundaries of the acute medical service in the context of the wider unscheduled care pathway. Similarly, national benchmarking that compares performance across units with the intent of improving overall care requires a robust definition to ensure like for like comparisons. Previous efforts at undertaking quality improvement across Scotland have been significantly challenged by variations in definitions of acute medical activity[24]. Identifying a clear study population based upon an evidence based definition is a prerequisite to research that aims to evaluate methods of care delivery. This work is much needed given the current lack of discerning evidence informing how best to deliver care in AMUs[15]. This recent systematic review, which asked how best to deliver care in AMUs, found the evidence to be limited in scope with the majority of it being single-centred and potentially confounded. The exception to this is the evidence relating to the potential benefit of increased consultant presence on the AMU. Onward research to develop this evidence base would involve comparisons of groups of patients that differ in their exposure to specific delivery models but are otherwise similar. This requires a universal method to define the study population, such as the one suggested in this paper, without which comparisons between groups become meaningless.

Secondly, we have delineated the key components of AMU care. This is of relevance operationally, as it offers a foundation on which acute medical care can be further considered and strategized, again at unit and policy level. From a research perspective, this framework provides an essential starting point for evaluating AMUs by providing a comprehensive structure for the design of future empirical investigations.

Strengths of this work are its combined inductive and deductive approach, the inclusion of a broad range of healthcare professionals across the multidisciplinary team, the verification of the data by unit representatives and the independent validation undertaken throughout the study. Furthermore, the universal sampling strategy mitigated any risk of omission in the data and ensured the full representation of the acute medicine community in Scotland. This study is limited by examining the AMU model from the perspective of healthcare professionals. Importantly, owing to resource constraints, there were no patients involved in the research process. The patient perspective on the provision of acute care is an important area for further research.

In summary, this empirically derived characterisation of the AMU model is likely to be of utility to practitioners, managers, policy makers and researchers: it is relevant on an operational level, will aid quality improvement and is a foundation to needed further research into how best to deliver care AMUs. This is important given the central role AMUs play in the journey of the majority of patients presenting to hospital acutely in Scotland, the UK and internationally.

## Supporting information

S1 checklist Consolidated criteria for reporting qualitative studies (COREQ): 32-item checklist. Developed from: Tong A, Sainsbury P, Craig J. Consolidated criteria for reporting qualitative research (COREQ): a 32-item checklist for interviews and focus groups. *International Journal for Quality in Health Care*. 2007. Volume 19, Number 6: pp. 349–357
(DOCX)
